# Response to the Toast, “Dentistry and Dental Education—Its past, Present and Future, as Related to Medicine”

**Published:** 1881-05

**Authors:** W. W. Allport


### RESPONSE TO THE TOAST, "DENTISTRY AND DENTAL EDUCATION—ITS PAST, PRESENT AND FUTURE, AS RELATED TO MEDICINE."

BY W. W. ALLPORT, M. D., D. D. S.

*Delivered before the Alumni Association of Rush Medical College, Chicago.*

I hardly know how to respond to the sentiment just read. "Dentistry and Dental Education—its Past, Present and Future, as related to medicine," contains, as the lawyers would say, several "distinct counts," either of which gives rise to more thought than can well be expressed in the time it would be proper for me to occupy on the present occasion. Contrary to the popular notion, dentistry is not entirely of modern origin. Its history dates far back into the centuries.

To respond fully, therefore, to the sentiment proposed, I should be obliged to trace carefully and consecutively its history, both ancient and modern, and assume to be a prophet and foretell its future.

With facts at hand and time to do it, it is not a difficult task to write history; but it is always a risky business to venture upon prophecy.

As we are far more interested in the present and future of dentistry than in the far-off past, I shall be pardoned, I trust, Mr. President, if I but briefly allude to its early history, and confine what I may say to more modern times; and instead of predicting the future of dentistry, I will state what I think it ought to be, trusting that what should be will be.

Of the early history of dentistry we know but little. That it was practiced at a very early period is fully proven by the facts that teeth well preserved by gold fillings have been found in the mouths of mummies, and also that artificial teeth, mounted upon gold plates have been discovered in the monuments of the Egyptians.

We are told that the ancients practiced it as a specialty in medicine. But if so, its practice—like that of surgery—seems to have fallen into the hands of mechanics at a later period.

During the latter part of what is known as the middle ages in Europe, surgery, as you are aware, was practiced by barbers, by the advice and under the direction of physicians; and it will hardly do to suppose that at this time dentistry was of higher rank than surgery.

Soon after this, or cotemporary with the period mentioned by medical writers as the revival of surgery, the first steps were taken toward modern dental surgery; and associated with the dental literature of those times stand the names of Eustachius, Fallopius and Pare, known to have been among the first, if they were not the very first, surgeons and writers of their times. In fact, it is owing to these great men of that day that surgery, whether general or special, was first placed upon a scientific and reliable foundation of dissection. Later, other medical men wrote more or less upon the anatomy, diseases and treatment of the teeth. Prominent among these was John Hunter, up to whose time England or the world had not produced so great a surgeon. I mention these things to remind you that the writings upon the teeth up to about the last of the eighteenth and first of the nineteenth centuries were by anatomists and surgeons, and, I will add, that they treated more of the structure, functions and diseases of the teeth than in giving any practical direction as to their treatment when diseased.

As the structure and functions of the teeth became better understood, and their importance to a healthy condition of the body more fully realized, medical men began to appreciate the importance of their preservation. But class influence, which has always ruled so strongly in European countries, and placed the ban of social ostracism upon all who engage in hand labor, may be assigned as one reason why practical dentistry did not receive its first great impulse from those who did so much to enlighten the medical profession as to the structure of the teeth and their diseases.

Dental operations at that time, whatever they may have been —like the rude surgery of a century or two before—seem to have been practiced as an art; the latter by barbers, and the former by jewelers and smiths. Dentistry in those days consisted very largely of making artificial teeth.

During the latter part of the eighteenth century, the art began to be practiced in America. As it proved to be remunerative for the services rendered, and as muscle and brain enter into a more equal contest for respectability in America than in Europe, our country became the most fruitful field for the development of practical dental science. Americans, always quick of perception, soon discovered that diseases of the teeth could not be intelligently and successfully treated without a knowledge of their anatomical structure, as well as of their surroundings; and like the barber-surgeons of whom I have spoken, they began to study anatomy. Some even ventured so far upon the domain of medicine as to acquire a limited knowledge of physiology and pathology, and, with the purchase of a few secret prescriptions, they assumed to be and the people dubbed them as "doctors." As the need for a more scientific education became apparent, medical men began to engage in the practice of dentistry, both as operators upon the natural teeth and as makers of artificial teeth. From this point, especially in our own country, the science and practice of dentistry rapidly developed. Dental magazines and text-books were published, dental associations and colleges were established, and soon the superior skill of American dentists became so generally acknowledged that their services were in demand in nearly all the countries of Europe.

When dental colleges were first established, dentistry consisted mainly in the cleaning and extraction of the natural teeth, in the treatment of inflamed or diseased gums and in filling what would now be regarded as the simpler forms of cavities; also in mounting artificial teeth upon gold and silver plates, in the setting of pivot teeth and in regulating the simpler cases of mal-arranged teeth. At that period, extraction was the approved treatment for teeth with exposed or aching pulps, abscess of roots or absorbed sockets. *Now*, not only teeth with exposed but dead pulps, abscess of roots, atrophied surroundings and necrosed jaws are regularly and successfully treated and saved. In no corresponding period of time have the improvements in surgery been so great as during the present century, and in no department of conservative surgery have they been so emphasized as in the treatment of diseases of the teeth and mouth; and to my calling must the credit of this improvement be mainly given. *Now* to adopt extraction as the rule of practice in such cases would be considered mal-practice.

Artificial teeth are now mounted not only upon gold and silver plates but upon at least ten different bases, of various degrees of applicability and usefulness in the cases requiring substitutes—each requiring an entirely different manipulation and mode of treatment. In order to bestow the greatest benefits of the art, a thorough knowledge of making and applying these various kinds of work is absolutely essential.

In addition to the great improvements in the setting of artificial teeth, as a branch of mechanical dentistry, artificial fixtures are now so constructed as to be far more generally useful in the treatment of cleft palate than is the surgeon's knife. These improvements are but indications of the advance in all directions in practice.

In the earlier days of our dental schools, so little was embraced within the practice of dentistry that a two-years' course of college instruction seemed sufficient to qualify students to practice in the two departments at the standard then maintained. But with the improvements and expansion thus far made, it is no longer possible. There is enough now embraced in either department to engage the entire time and talent of any one individual, and the greatest excellence can only be secured by a division of practice. Those whose tastes and talents best fit them for mechanical dentistry should pursue that as a branch of art, and give it their exclusive attention; and those who practice dental and oral surgery should pursue it as a branch of medicine, and educate themselves accordingly. To this division of practice the objection is urged that the two departments "lock and inter-lock" so intimately that the separation is not practicable. No doubt patients would find it more convenient to receive the mechanical and medical treatment from the same person. So, too, it would be more convenient for the afflicted to have their limbs amputated and artificial ones supplied by the same practitioner; or to have the otologist treat the ear and furnish ear-trumpets of his own manufacture. But experience has proven that patients are best served by receiving medical treatment from medical men, and manufactured articles from mechanical artists.

The same principle holds good, and must hold good, in the two departments of dentistry.

An accomplished mechanical dentist must now not only understand the nature and manner of manipulating the various materials I have mentioned into plates for artificial teeth, but he must be so skilled as a mechanic that he will be able to adapt these materials to the requirements of the case in hand in such a way that patients will derive the greatest benefit possible from artificial teeth as masticators. He should be so versed in art that he can adapt them to the face of the patient—in size, shape, color and position in the mouth—so perfectly as to conceal the fact that they are not natural; and so fully should he understand the essentials to correct and easy enunciation that they will act as aids instead of impediments to speech, as is now too frequently the case.

The apprenticeship to an ordinary trade is not less than three years, and it can hardly be claimed that less time should be devoted to acquiring this most difficult art than is spent in learning to be a blacksmith or a carpenter. Whoever pursues the calling properly will have no time for the practice of medicine in any of its branches.

About the same degree of anatomical knowledge should be required of the mechanical dentist that is expected of the painter, sculptor or maker of artificial limbs, and more than this is scarcely necessary.

I have now, as concisely as possible, given you a general idea of the history and present status of dentistry; and I wish to say, in as emphatic a manner as I can, that it is to our dental colleges, and to the arduous and self-denying labors of the teachers in those schools, more than to any other cause, that dentistry owes its present honorable position in the community and its standing among the sciences. Each succeeding year the teachings in our dental colleges are becoming more scientific, reaching farther into the realm of medicine; and still they claim to cover but a portion of the science in their teaching. In proportion as students are inclined to the study of medicine are they disinclined to practice in mechanical dentistry. Not only is it becoming distasteful to this class of practitioners, but they are dissatisfied with confining their operations exclusively to the teeth. Gradually but surely are the best medically educated dentists reaching for, and bringing within the scope of their practice all operations and treatment not only of the teeth but of the jaws and oral cavity, and owing to their great familiarity with the diseases of the mouth and their habitual use of the delicate instruments required, it is fitting that medically educated specialists in dental and oral surgery should control this department of practice. The ease and accuracy they acquire in operating about these parts could not be possessed by the general surgeon.

Any treatment for arresting disease is legitimately a branch of medicine, no matter whether it be pills, powders, the surgeon's knife, the cautery or materials for filling teeth. All are equally therapeutical agents. But their intelligent use must be based upon a knowledge of anatomy, physiology, pathology, chemistry and therapeutics; for the human organism is so intimately related that each part either directly or indirectly, affects every other part. The same heart's blood sends its arterial currents to every point; the same nerve centers radiate their impressions throughout the system, and the same vital force pervades every atom of its structure. This specialism, therefore, cannot be legitimate unless its practice is laid in the fundamental principles of medicine.

Appreciating this fact, there is a growing tendency among the best dental graduates to make the diseases of the teeth and mouth a specialty in medical practice; and that they may be better qualified to do this, many of them supplement their dental education with a full course of instruction at our medical colleges.

Much of this time could be saved if these colleges provided ample instruction in dental and oral surgery, and exacted of their graduates a knowledge of the pathology of the diseases in this specialty and the science of their treatment.

Dental text-books devoted to diseases and treatment of the teeth are seldom found in medical libraries, and medical text-books, as well as lectures in medical colleges, have hitherto been comparatively silent upon this subject; which fact has necessitated the organization and maintainance of dental colleges—until it is claimed by some that dentistry has almost created for itself a separate science. But since the teeth constitute a portion of the human body, there can be no separate science for the treatment of their diseases.

All change from rest to unrest in any organ of the body is a change from health to disease, and its pathology and treatment should be taught in medical text-books and colleges. Without it they are but partial teachers of the curative art, and fall, therefore, short of accomplishing their whole mission. As medical colleges are now constituted, medical graduates go forth in as great or greater ignorance of diseases of the teeth than do dental graduates of general disease. As an illustration of this lack of knowledge on the part of the physicians, I will give a single instance, which is a fair sample of others constantly occuring, and which could be given by almost any dental practitioner.

A physician of large and respectable practice came to me a few months ago with a patient having a swollen face. Said he, "Doctor, this patient has been suffering for several days with toothache, and as nothing I can do affords relief I have brought her to you to have the nerve killed." I replied:

"Yes, Doctor, I see it is an ulcerated tooth. The nerve has undoubtedly been dead for a long time."

On raising the upper lip, I called his attention to an abscess just ready to burst, over the root of a cuspid tooth. I took a bistoury and, with a slight puncture, brought the pus. Said he, "Doctor, are you *sure* the nerve in that tooth is dead?" I replied that an abscess over the root of a tooth was one of the surest indications of the death of the pulp, and passed an instrument the entire length of the canal, to convince him that the pulp was dead. He looked thoughtful for a moment, and said, "I don't think physicians know much about diseases of the teeth." I told him I thought he was nearer right in that remark than in his diagnosis of this case.

Now, this physician was not only a professor in a medical college but he was the occupant of a chair, which made it reasonable to suppose that he would know the difference between pulpitis and alveolar abscess. Had he but listened to a few lectures on dental pathology, in his student days, he would hardly have made this humiliating mistake.

Medical colleges do not educate specialists; they but lay the foundation for general practice. This knowledge acquired, the practitioner may turn his attention to general practice or the specialty of his choice; and by the application of the principles first taught, he is supposed to become more than ordinarily skilled in the treatment of the particular class of diseases in his specialty.

With this idea in view, there is no more reason why we should have colleges for the teaching of the science involved in dental or oral surgery, independently of general medicine, than for general surgery.

One of the first requisites to success in the practice of any department of surgery or dentistry, is mechanical and executive talent. Without these a physician may be able to diagnosticate accurately and prescribe scientifically, but he can never succeed as an operator, either in dental or general surgery. But if he is educated in either of these specialties, without a general medical education, he is left no choice save to follow that branch for which he is educated, though he finds by experience that he has not the requisite talent to succeed in its practice. Many graduates of our dental colleges find themselves in this very unpleasant position. The result is, we have too many engaged in dental practice who, neither by taste nor talent, are fitted for it; and, lacking a medical education, they cannot engage in general practice. Properly educated, many of them would make excellent practitioners in medicine, but they are failures as dentists, as they would have been as surgeons.

The change advocated would give the student a chance for the exercise of his choice, enabling him, after his regular college course, to select the special branch for which he feels himself to be best adapted. If he chooses the specialty of dental and oral surgery, rather than to engage in the general practice of medicine, the teaching he has received should be supplemented by such clinical and other instruction as will best qualify him to engage in that department of practice. A medical education should embrace a general knowledge of all diseases to which the human organism is liable, and their treatment. Specialism includes this knowledge, to which is added careful and special study of the diseases and treatment that come within a given department of practice. A dental education, therefore, is a medical education, *plus* dental—or, special knowledge of the diseases and treatment of the teeth and oral cavity.

I therefore advocate the teaching in our medical colleges of the science of dental and oral surgery, not only that dental students may gain a broader medical education, but that medical students may be better informed regarding the diseases and treatment of the teeth. The signature of the professor or professors of dental and oral surgery should be considered as important upon the diploma of graduation as the signatures of the teachers in any other department.

At no previous time in the history of medicine in our country has the necessity for a fuller course of medical instruction become so apparent as the present; and it will not be long before a three-years course will be requisite to the respectable standing of any medical college with the profession. In making this change the fact should not be overlooked that their course cannot be complete without the teaching of the diseases and treatment of the teeth by properly educated and practical dental and oral surgeons.

With this broader and better medical education on the part of dental and oral surgeons, and a better knowledge of the nature and influence of the diseases of the teeth and mouth, and their proper treatment, on the part of medical men, there could be no valid objection to the establishment of a section in dental and oral surgery in the American Medical Association, upon an equal footing with any other department of medical science. The importance of this movement is being discussed among the most advanced dental practitioners and the most comprehensive medical teachers. Sooner or later, this fuller and better education and condition must come, and the action of our medical colleges will largely determine whether its consumation will be hastened or retarded; but though delayed, this ultimate result will obtain.

As America, less wedded to old ideas and practices than Europe, gave to practical dentistry its first great impulse, so, too, it may not be impossible that in this great Northwest, where advanced ideas more rapidly take root and expand, and new needs are more readily met, the first steps may be taken towards this desired end.

Having detained you longer than is customary on such occasions, with my thanks for your kind attention, and believing that some such plan as I have suggested for dental and medical instruction would be beneficial to the public, as well as those engaged in practice, I leave these thoughts for your consideration. If, perchance, what I have said should receive a greater audience than this presence, I ask for it considerate thought and a generous criticism.

